# 3,5-Dimethyl-1*H*-pyrazole–2-hy­droxy-5-(phenyl­diazen­yl)benzoic acid (1/1)

**DOI:** 10.1107/S1600536811034970

**Published:** 2011-08-31

**Authors:** Yichao Xu, Shouwen Jin, Jianlong Zhu, Ying-Jia Liu, Chuan-Chuan Shi

**Affiliations:** a Tianmu college of ZheJiang A & F University, Lin’An 311300, People’s Republic of China

## Abstract

There are two independent 3,5-dimethyl­pyrazole and two independent 2-hy­droxy-5-(phenyl­diazen­yl)benzoic acid mol­ecules [in which intra­molecular O—H⋯O bonds form *S*(6) graph-set motifs] in the asymmetric unit of the title compound, C_5_H_8_N_2_·C_13_H_10_N_2_O_3_. In the crystal, the components are linked by inter­molecular O—H⋯O, O—H⋯N and N—H⋯O hydrogen bonds, forming four-component clusters. Further stabilization is provided by weak C—H⋯π inter­actions.

## Related literature

For general background to hydrogen-bonding inter­actions, see: Lam & Mak (2000 ▶); Desiraju (2002 ▶); Liu *et al.* (2008 ▶); Biswas *et al.* (2009 ▶); Jin *et al.* (2010 ▶). For hydrogen-bond motifs, see: Bernstein *et al.* (1995 ▶).
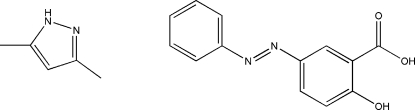


## Experimental

### 

#### Crystal data

C_5_H_8_N_2_·C_13_H_10_N_2_O_3_
*M* *_r_* = 338.36
Triclinic, 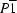

*a* = 11.4871 (11) Å
*b* = 12.4746 (13) Å
*c* = 13.2235 (16) Å
α = 86.650 (2)°
β = 67.540 (1)°
γ = 77.715 (1)°
*V* = 1710.5 (3) Å^3^
*Z* = 4
Mo *K*α radiation
μ = 0.09 mm^−1^
*T* = 298 K
0.38 × 0.28 × 0.20 mm

#### Data collection

Bruker SMART CCD diffractometer
Absorption correction: multi-scan (*SADABS*; Bruker, 2002 ▶) *T*_min_ = 0.970, *T*_max_ = 0.982
8928 measured reflections
5945 independent reflections
2614 reflections with *I* > 2σ(*I*)
*R*_int_ = 0.025

#### Refinement

*R*[*F* ^2^ > 2σ(*F* ^2^)] = 0.050
*wR*(*F* ^2^) = 0.159
*S* = 0.99
5945 reflections
455 parameters
H-atom parameters constrained
Δρ_max_ = 0.21 e Å^−3^
Δρ_min_ = −0.17 e Å^−3^

### 

Data collection: *SMART* (Bruker, 2002 ▶); cell refinement: *SAINT* (Bruker, 2002 ▶); data reduction: *SAINT*; program(s) used to solve structure: *SHELXS97* (Sheldrick, 2008 ▶); program(s) used to refine structure: *SHELXL97* (Sheldrick, 2008 ▶); molecular graphics: *SHELXTL* (Sheldrick, 2008 ▶) and *Mercury* (Macrae *et al.*, 2006 ▶); software used to prepare material for publication: *SHELXL97*.

## Supplementary Material

Crystal structure: contains datablock(s) global, I. DOI: 10.1107/S1600536811034970/lh5315sup1.cif

Structure factors: contains datablock(s) I. DOI: 10.1107/S1600536811034970/lh5315Isup2.hkl

Supplementary material file. DOI: 10.1107/S1600536811034970/lh5315Isup3.cml

Additional supplementary materials:  crystallographic information; 3D view; checkCIF report

## Figures and Tables

**Table 1:** Hydrogen-bond geometry (Å, °) *Cg*1 and *Cg*2 are the centroids of the C31–C36 and C25–C30 rings, respectively.

*D*—H⋯*A*	*D*—H	H⋯*A*	*D*⋯*A*	*D*—H⋯*A*
O6—H6⋯O2^i^	0.82	2.43	3.019 (3)	129
O6—H6⋯O5	0.82	1.89	2.605 (3)	145
O4—H4*A*⋯N4^ii^	0.82	1.75	2.564 (3)	169
O3—H3*A*⋯O5^iii^	0.82	2.45	3.019 (3)	128
O3—H3*A*⋯O2	0.82	1.85	2.574 (3)	147
O1—H1*A*⋯N1^iv^	0.82	1.75	2.565 (3)	171
N3—H3⋯O3^v^	0.86	2.17	3.008 (3)	165
N2—H2⋯O6^vi^	0.86	2.15	3.002 (3)	170
C1—H1*D*⋯*Cg*1^vii^	0.96	2.75	3.677 (4)	164
C5—H5*B*⋯*Cg*2^vii^	0.96	2.96	3.840 (4)	153

Symmetry codes: (i) 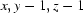; (ii) 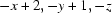; (iii) 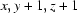; (iv) 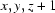; (v) 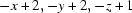; (vi) 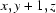; (vii) 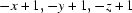.

**Fig. 1.**
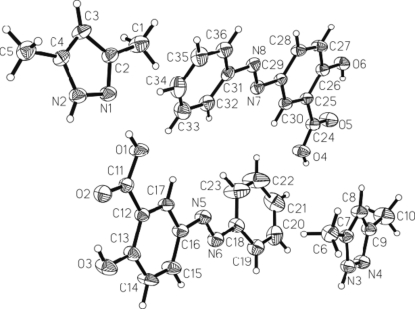
The asymmetric unit of the title compound. Displacement ellipsoids are drawn at the 30% probability level.

**Fig. 2.**
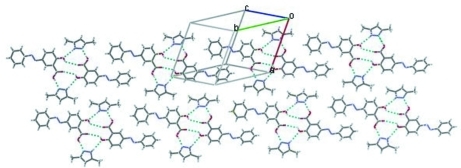
Part of the crystal structure with hydrogen bonds shown as dashed lines.

## supplementary crystallographic information

### Comment

Intermolecular interactions are responsible for crystal packing and gaining an understanding of these interactions allows the comprehension of the collective properties and permits the design of new crystals with specific physical and chemical properties (Lam & Mak, 2000). Hydrogen bonding is one of the most important noncovalent interactions that determines and controls the assembly of molecules and ions (Desiraju, 2002, Liu *et al.*, 2008, Biswas *et al.*, 2009). As an extension of our study concentrating on hydrogen bonded assembly of organic acids and organic bases (Jin *et al.*, 2010), herein we report the crystal structure of the 1:1 adduct of 3,5-dimethyl pyrazole and 2-hydroxy-5-(phenyldiazenyl)benzoic acid.

The asymmetric unit of the compound consists of two independent 3,5-dimethyl pyrazole and two independent 2-hydroxy-5-(phenyldiazenyl)benzoic acid molecules (Fig. 1). Intramolecular hydrogen bonds between the phenol O—H groups and the carbonyl groups form S(6) graph motifs (Bernstein *et al.*, 1995). The two independent carboxylic acid molecules form a dimer through O—H···O hydrogen bonds in which the phenol group is the donor and the carbonyl O atom acts as the acceptor. The two pyrazole molecules are linked to the carboxylic dimer through the N—H···O, and O—H···N hydrogen bonds to form a four component adduct containing *R*_2_^2^(12) and *R*_3_^3^(9) ring motifs (Fig. 2). Further stabilization is provided by weak C—H···π interactions.

### Experimental

A solution of 3,5-dimethyl pyrazole (19.2 mg, 0.2 mmol) in 5 ml of MeOH was added to a MeOH solution (3 ml) containing 2-hydroxy-5-(phenyldiazenyl)benzoic acid (48.4 mg, 0.2 mmol) under continuous stirring. The solution was stirred for about 1 h at room temperature, then the solution was filtered into a test tube. The solution was left standing at room temperature for several days, red block-shaped crystals were isolated after slow evaporation of the solution in air at ambient temperature.

### Refinement

All H atoms were placed in calculated positions with C—H = 0.93-0.96 Å, N—H = 0.86Å and O—H = 0.82Å and were included in the refinement with U_iso_(H)=1.2U_eq_(C,N) or U_iso_(H)=1.2U_eq_(C_methyl_,O).

### Crystal data

**Table:**

C_5_H_8_N_2_·C_13_H_10_N_2_O_3_	*Z* = 4
*M**_r_* = 338.36	*F*(000) = 712
Triclinic, *P*1	*D*_x_ = 1.314 Mg m^−^^3^
Hall symbol: -P 1	Mo *K*α radiation, λ = 0.71073 Å
*a* = 11.4871 (11) Å	Cell parameters from 1438 reflections
*b* = 12.4746 (13) Å	θ = 2.4–21.4°
*c* = 13.2235 (16) Å	µ = 0.09 mm^−^^1^
α = 86.650 (2)°	*T* = 298 K
β = 67.540 (1)°	Block, red
γ = 77.715 (1)°	0.38 × 0.28 × 0.20 mm
*V* = 1710.5 (3) Å^3^	

### Data collection

**Table:**

Bruker SMART CCD diffractometer	5945 independent reflections
Radiation source: fine-focus sealed tube	2614 reflections with *I* > 2σ(*I*)
graphite	*R*_int_ = 0.025
φ and ω scans	θ_max_ = 25.0°, θ_min_ = 2.4°
Absorption correction: multi-scan (*SADABS*; Bruker, 2002)	*h* = −13→13
*T*_min_ = 0.970, *T*_max_ = 0.982	*k* = −14→14
8928 measured reflections	*l* = −15→12

### Refinement

**Table:**

Refinement on *F*^2^	Primary atom site location: structure-invariant direct methods
Least-squares matrix: full	Secondary atom site location: difference Fourier map
*R*[*F*^2^ > 2σ(*F*^2^)] = 0.050	Hydrogen site location: inferred from neighbouring sites
*wR*(*F*^2^) = 0.159	H-atom parameters constrained
*S* = 0.99	*w* = 1/[σ^2^(*F*_o_^2^) + (0.0639*P*)^2^] where *P* = (*F*_o_^2^ + 2*F*_c_^2^)/3
5945 reflections	(Δ/σ)_max_ < 0.001
455 parameters	Δρ_max_ = 0.21 e Å^−^^3^
0 restraints	Δρ_min_ = −0.17 e Å^−^^3^

### Special details

**Table:**

Geometry. All e.s.d.'s (except the e.s.d. in the dihedral angle between two l.s. planes) are estimated using the full covariance matrix. The cell e.s.d.'s are taken into account individually in the estimation of e.s.d.'s in distances, angles and torsion angles; correlations between e.s.d.'s in cell parameters are only used when they are defined by crystal symmetry. An approximate (isotropic) treatment of cell e.s.d.'s is used for estimating e.s.d.'s involving l.s. planes.
Refinement. Refinement of *F*^2^ against ALL reflections. The weighted *R*-factor *wR* and goodness of fit *S* are based on *F*^2^, conventional *R*-factors *R* are based on *F*, with *F* set to zero for negative *F*^2^. The threshold expression of *F*^2^ > σ(*F*^2^) is used only for calculating *R*-factors(gt) *etc*. and is not relevant to the choice of reflections for refinement. *R*-factors based on *F*^2^ are statistically about twice as large as those based on *F*, and *R*- factors based on ALL data will be even larger.

### Fractional atomic coordinates and isotropic or equivalent isotropic displacement parameters (Å^2^)

**Table:**

	*x*	*y*	*z*	*U*_iso_*/*U*_eq_	
N1	0.4488 (2)	0.8896 (2)	0.1548 (2)	0.0495 (7)	
N2	0.4409 (2)	0.9469 (2)	0.2418 (2)	0.0517 (7)	
H2	0.4842	0.9967	0.2372	0.062*	
N3	0.8917 (3)	0.8335 (2)	0.1146 (2)	0.0589 (8)	
H3	0.9400	0.8778	0.1139	0.071*	
N4	0.9161 (3)	0.7637 (2)	0.0310 (2)	0.0627 (8)	
N5	0.8889 (2)	0.7607 (2)	0.5787 (2)	0.0552 (8)	
N6	0.9694 (2)	0.7542 (2)	0.4833 (2)	0.0556 (8)	
N7	0.6155 (3)	0.3798 (2)	0.5330 (2)	0.0520 (7)	
N8	0.5186 (3)	0.4064 (2)	0.6184 (2)	0.0538 (7)	
O1	0.61956 (19)	0.87430 (17)	0.95957 (16)	0.0598 (7)	
H1A	0.5662	0.8861	1.0223	0.090*	
O2	0.7063 (2)	0.99484 (18)	1.01085 (16)	0.0576 (6)	
O3	0.9136 (2)	1.04618 (19)	0.87199 (18)	0.0731 (8)	
H3A	0.8553	1.0448	0.9318	0.110*	
O4	0.8984 (2)	0.25974 (19)	0.15689 (17)	0.0732 (8)	
H4A	0.9552	0.2444	0.0962	0.110*	
O5	0.8065 (2)	0.1510 (2)	0.09796 (19)	0.0806 (8)	
O6	0.5691 (2)	0.13862 (17)	0.21526 (17)	0.0638 (7)	
H6	0.6370	0.1266	0.1618	0.096*	
C1	0.3553 (3)	0.7443 (3)	0.1208 (3)	0.0678 (11)	
H1B	0.3986	0.7633	0.0464	0.102*	
H1C	0.2657	0.7498	0.1353	0.102*	
H1D	0.3928	0.6705	0.1323	0.102*	
C2	0.3689 (3)	0.8209 (3)	0.1960 (3)	0.0491 (8)	
C3	0.3104 (3)	0.8361 (3)	0.3096 (2)	0.0548 (9)	
H3B	0.2508	0.7982	0.3576	0.066*	
C4	0.3572 (3)	0.9167 (3)	0.3362 (2)	0.0492 (9)	
C5	0.3317 (3)	0.9694 (3)	0.4430 (2)	0.0640 (10)	
H5A	0.4039	0.9999	0.4373	0.096*	
H5B	0.3187	0.9156	0.4989	0.096*	
H5C	0.2559	1.0268	0.4615	0.096*	
C6	0.7346 (3)	0.8921 (3)	0.3023 (3)	0.0678 (10)	
H6A	0.7835	0.8618	0.3459	0.102*	
H6B	0.6453	0.8916	0.3427	0.102*	
H6C	0.7447	0.9662	0.2846	0.102*	
C7	0.7820 (3)	0.8248 (3)	0.1991 (3)	0.0526 (9)	
C8	0.7350 (3)	0.7468 (3)	0.1691 (3)	0.0580 (10)	
H8	0.6600	0.7225	0.2106	0.070*	
C9	0.8199 (3)	0.7099 (3)	0.0644 (3)	0.0576 (9)	
C10	0.8155 (4)	0.6257 (3)	−0.0085 (3)	0.0884 (13)	
H10A	0.8595	0.6427	−0.0835	0.133*	
H10B	0.7275	0.6254	0.0044	0.133*	
H10C	0.8569	0.5548	0.0066	0.133*	
C11	0.7069 (3)	0.9320 (3)	0.9424 (2)	0.0466 (8)	
C12	0.8086 (3)	0.9170 (2)	0.8312 (2)	0.0421 (8)	
C13	0.9061 (3)	0.9767 (3)	0.8012 (3)	0.0527 (9)	
C14	0.9967 (3)	0.9671 (3)	0.6950 (3)	0.0680 (11)	
H14	1.0596	1.0091	0.6743	0.082*	
C15	0.9950 (3)	0.8972 (3)	0.6202 (3)	0.0606 (10)	
H15	1.0573	0.8911	0.5495	0.073*	
C16	0.9007 (3)	0.8348 (3)	0.6493 (2)	0.0489 (8)	
C17	0.8081 (3)	0.8470 (2)	0.7541 (2)	0.0473 (8)	
H17	0.7435	0.8070	0.7734	0.057*	
C18	0.9564 (3)	0.6770 (3)	0.4144 (3)	0.0529 (9)	
C19	1.0231 (3)	0.6814 (3)	0.3045 (3)	0.0679 (11)	
H19	1.0742	0.7331	0.2772	0.081*	
C20	1.0147 (4)	0.6087 (4)	0.2336 (3)	0.0809 (12)	
H20	1.0599	0.6118	0.1586	0.097*	
C21	0.9407 (4)	0.5330 (4)	0.2733 (4)	0.0923 (14)	
H21	0.9358	0.4834	0.2260	0.111*	
C22	0.8738 (5)	0.5302 (4)	0.3826 (4)	0.127 (2)	
H22	0.8214	0.4793	0.4099	0.152*	
C23	0.8823 (4)	0.6011 (3)	0.4529 (3)	0.1058 (17)	
H23	0.8370	0.5973	0.5277	0.127*	
C24	0.8065 (3)	0.2099 (3)	0.1692 (3)	0.0570 (9)	
C25	0.6965 (3)	0.2315 (2)	0.2758 (2)	0.0465 (8)	
C26	0.5819 (3)	0.1987 (2)	0.2917 (3)	0.0483 (8)	
C27	0.4752 (3)	0.2303 (3)	0.3866 (3)	0.0560 (9)	
H27	0.3980	0.2110	0.3956	0.067*	
C28	0.4815 (3)	0.2895 (3)	0.4676 (2)	0.0528 (9)	
H28	0.4090	0.3099	0.5312	0.063*	
C29	0.5965 (3)	0.3193 (2)	0.4548 (3)	0.0468 (8)	
C30	0.7017 (3)	0.2909 (2)	0.3588 (2)	0.0488 (8)	
H30	0.7780	0.3121	0.3493	0.059*	
C31	0.5405 (3)	0.4670 (2)	0.6963 (2)	0.0496 (9)	
C32	0.6597 (4)	0.4840 (3)	0.6864 (3)	0.0624 (10)	
H32	0.7329	0.4540	0.6266	0.075*	
C33	0.6705 (4)	0.5451 (3)	0.7646 (3)	0.0713 (11)	
H33	0.7504	0.5575	0.7569	0.086*	
C34	0.5632 (4)	0.5873 (3)	0.8538 (3)	0.0774 (12)	
H34	0.5703	0.6280	0.9071	0.093*	
C35	0.4450 (4)	0.5699 (3)	0.8646 (3)	0.0725 (11)	
H35	0.3724	0.5992	0.9252	0.087*	
C36	0.4329 (3)	0.5094 (3)	0.7868 (3)	0.0613 (10)	
H36	0.3527	0.4972	0.7951	0.074*	

### Atomic displacement parameters (Å^2^)

**Table:**

	*U*^11^	*U*^22^	*U*^33^	*U*^12^	*U*^13^	*U*^23^
N1	0.0481 (16)	0.0578 (18)	0.0385 (16)	−0.0182 (14)	−0.0075 (13)	−0.0027 (14)
N2	0.0525 (17)	0.0597 (18)	0.0410 (17)	−0.0227 (14)	−0.0094 (14)	−0.0031 (14)
N3	0.0597 (19)	0.073 (2)	0.0483 (18)	−0.0304 (16)	−0.0164 (16)	0.0005 (16)
N4	0.0631 (19)	0.079 (2)	0.0451 (18)	−0.0292 (17)	−0.0107 (15)	−0.0092 (16)
N5	0.0552 (17)	0.0649 (19)	0.0401 (17)	−0.0225 (15)	−0.0053 (15)	−0.0099 (14)
N6	0.0554 (18)	0.0650 (19)	0.0389 (17)	−0.0170 (15)	−0.0061 (15)	−0.0080 (14)
N7	0.0613 (18)	0.0526 (17)	0.0398 (17)	−0.0159 (14)	−0.0140 (15)	−0.0031 (14)
N8	0.0633 (18)	0.0515 (17)	0.0436 (18)	−0.0136 (15)	−0.0154 (16)	−0.0032 (14)
O1	0.0581 (14)	0.0766 (16)	0.0376 (13)	−0.0327 (13)	0.0008 (11)	−0.0085 (11)
O2	0.0603 (15)	0.0695 (16)	0.0400 (13)	−0.0207 (12)	−0.0098 (11)	−0.0132 (12)
O3	0.0655 (15)	0.0945 (19)	0.0567 (16)	−0.0406 (14)	−0.0035 (13)	−0.0268 (14)
O4	0.0658 (16)	0.0992 (19)	0.0498 (15)	−0.0391 (15)	−0.0022 (13)	−0.0172 (13)
O5	0.0840 (18)	0.101 (2)	0.0523 (16)	−0.0412 (16)	−0.0049 (14)	−0.0297 (14)
O6	0.0666 (15)	0.0741 (16)	0.0534 (15)	−0.0312 (13)	−0.0141 (12)	−0.0171 (12)
C1	0.081 (3)	0.065 (2)	0.051 (2)	−0.024 (2)	−0.010 (2)	−0.0082 (18)
C2	0.056 (2)	0.049 (2)	0.041 (2)	−0.0153 (17)	−0.0139 (17)	0.0006 (16)
C3	0.065 (2)	0.052 (2)	0.038 (2)	−0.0221 (18)	−0.0052 (18)	0.0043 (16)
C4	0.053 (2)	0.053 (2)	0.035 (2)	−0.0107 (17)	−0.0085 (17)	0.0008 (16)
C5	0.076 (2)	0.067 (2)	0.047 (2)	−0.018 (2)	−0.0182 (19)	−0.0062 (18)
C6	0.070 (2)	0.073 (3)	0.056 (2)	−0.011 (2)	−0.018 (2)	−0.0111 (19)
C7	0.053 (2)	0.058 (2)	0.045 (2)	−0.0157 (18)	−0.0140 (18)	0.0003 (17)
C8	0.050 (2)	0.066 (2)	0.053 (2)	−0.0246 (18)	−0.0064 (18)	−0.0033 (18)
C9	0.059 (2)	0.065 (2)	0.050 (2)	−0.0225 (19)	−0.0167 (19)	−0.0048 (18)
C10	0.092 (3)	0.099 (3)	0.071 (3)	−0.036 (3)	−0.014 (2)	−0.029 (2)
C11	0.049 (2)	0.054 (2)	0.037 (2)	−0.0130 (18)	−0.0145 (17)	−0.0024 (17)
C12	0.0398 (18)	0.0488 (19)	0.0351 (18)	−0.0133 (15)	−0.0085 (15)	−0.0032 (15)
C13	0.050 (2)	0.062 (2)	0.048 (2)	−0.0189 (18)	−0.0142 (18)	−0.0120 (17)
C14	0.053 (2)	0.084 (3)	0.060 (2)	−0.041 (2)	0.004 (2)	−0.019 (2)
C15	0.048 (2)	0.075 (3)	0.049 (2)	−0.0275 (19)	0.0019 (18)	−0.0128 (19)
C16	0.0465 (19)	0.054 (2)	0.040 (2)	−0.0152 (17)	−0.0064 (17)	−0.0090 (16)
C17	0.0467 (19)	0.053 (2)	0.041 (2)	−0.0220 (16)	−0.0085 (17)	−0.0013 (16)
C18	0.047 (2)	0.058 (2)	0.046 (2)	−0.0089 (17)	−0.0081 (17)	−0.0142 (18)
C19	0.067 (2)	0.082 (3)	0.044 (2)	−0.020 (2)	−0.005 (2)	−0.012 (2)
C20	0.093 (3)	0.096 (3)	0.048 (2)	−0.004 (3)	−0.025 (2)	−0.021 (2)
C21	0.083 (3)	0.102 (4)	0.087 (4)	−0.019 (3)	−0.019 (3)	−0.048 (3)
C22	0.142 (4)	0.140 (4)	0.081 (4)	−0.090 (4)	0.014 (3)	−0.048 (3)
C23	0.135 (4)	0.106 (4)	0.058 (3)	−0.075 (3)	0.013 (3)	−0.031 (2)
C24	0.062 (2)	0.064 (2)	0.046 (2)	−0.023 (2)	−0.0150 (19)	−0.0086 (18)
C25	0.052 (2)	0.049 (2)	0.0391 (19)	−0.0183 (16)	−0.0127 (17)	−0.0016 (16)
C26	0.057 (2)	0.048 (2)	0.044 (2)	−0.0199 (17)	−0.0167 (18)	−0.0067 (16)
C27	0.055 (2)	0.059 (2)	0.054 (2)	−0.0256 (18)	−0.0110 (19)	−0.0089 (18)
C28	0.056 (2)	0.055 (2)	0.042 (2)	−0.0190 (18)	−0.0075 (17)	−0.0059 (17)
C29	0.055 (2)	0.046 (2)	0.041 (2)	−0.0146 (17)	−0.0171 (18)	−0.0020 (16)
C30	0.052 (2)	0.052 (2)	0.043 (2)	−0.0195 (17)	−0.0147 (18)	−0.0019 (16)
C31	0.071 (2)	0.045 (2)	0.036 (2)	−0.0153 (18)	−0.0224 (19)	0.0015 (16)
C32	0.072 (3)	0.063 (2)	0.050 (2)	−0.018 (2)	−0.018 (2)	−0.0057 (18)
C33	0.079 (3)	0.079 (3)	0.064 (3)	−0.023 (2)	−0.030 (2)	−0.012 (2)
C34	0.099 (3)	0.077 (3)	0.062 (3)	−0.006 (3)	−0.041 (3)	−0.022 (2)
C35	0.083 (3)	0.077 (3)	0.052 (2)	−0.007 (2)	−0.022 (2)	−0.022 (2)
C36	0.067 (2)	0.062 (2)	0.050 (2)	−0.0120 (19)	−0.017 (2)	−0.0081 (18)

### Geometric parameters (Å, °)

**Table:**

N1—C2	1.331 (3)	C10—H10B	0.9600
N1—N2	1.353 (3)	C10—H10C	0.9600
N2—C4	1.345 (3)	C11—C12	1.480 (4)
N2—H2	0.8600	C12—C17	1.384 (4)
N3—C7	1.348 (4)	C12—C13	1.395 (4)
N3—N4	1.353 (3)	C13—C14	1.385 (4)
N3—H3	0.8600	C14—C15	1.365 (4)
N4—C9	1.333 (4)	C14—H14	0.9300
N5—N6	1.242 (3)	C15—C16	1.390 (4)
N5—C16	1.413 (4)	C15—H15	0.9300
N6—C18	1.430 (4)	C16—C17	1.379 (4)
N7—N8	1.245 (3)	C17—H17	0.9300
N7—C29	1.420 (4)	C18—C23	1.355 (4)
N8—C31	1.436 (4)	C18—C19	1.363 (4)
O1—C11	1.301 (3)	C19—C20	1.385 (5)
O1—H1A	0.8200	C19—H19	0.9300
O2—C11	1.228 (3)	C20—C21	1.353 (5)
O3—C13	1.351 (3)	C20—H20	0.9300
O3—H3A	0.8200	C21—C22	1.356 (5)
O4—C24	1.290 (4)	C21—H21	0.9300
O4—H4A	0.8200	C22—C23	1.363 (5)
O5—C24	1.228 (4)	C22—H22	0.9300
O6—C26	1.362 (3)	C23—H23	0.9300
O6—H6	0.8200	C24—C25	1.482 (4)
C1—C2	1.490 (4)	C25—C30	1.385 (4)
C1—H1B	0.9600	C25—C26	1.397 (4)
C1—H1C	0.9600	C26—C27	1.382 (4)
C1—H1D	0.9600	C27—C28	1.368 (4)
C2—C3	1.397 (4)	C27—H27	0.9300
C3—C4	1.357 (4)	C28—C29	1.395 (4)
C3—H3B	0.9300	C28—H28	0.9300
C4—C5	1.490 (4)	C29—C30	1.377 (4)
C5—H5A	0.9600	C30—H30	0.9300
C5—H5B	0.9600	C31—C36	1.380 (4)
C5—H5C	0.9600	C31—C32	1.386 (4)
C6—C7	1.495 (4)	C32—C33	1.377 (4)
C6—H6A	0.9600	C32—H32	0.9300
C6—H6B	0.9600	C33—C34	1.369 (5)
C6—H6C	0.9600	C33—H33	0.9300
C7—C8	1.352 (4)	C34—C35	1.372 (5)
C8—C9	1.390 (4)	C34—H34	0.9300
C8—H8	0.9300	C35—C36	1.376 (4)
C9—C10	1.487 (4)	C35—H35	0.9300
C10—H10A	0.9600	C36—H36	0.9300
			
C2—N1—N2	105.5 (2)	C15—C14—H14	119.5
C4—N2—N1	111.8 (3)	C13—C14—H14	119.5
C4—N2—H2	124.1	C14—C15—C16	120.2 (3)
N1—N2—H2	124.1	C14—C15—H15	119.9
C7—N3—N4	111.4 (3)	C16—C15—H15	119.9
C7—N3—H3	124.3	C17—C16—C15	118.7 (3)
N4—N3—H3	124.3	C17—C16—N5	116.2 (3)
C9—N4—N3	105.6 (3)	C15—C16—N5	125.1 (3)
N6—N5—C16	115.6 (3)	C16—C17—C12	122.0 (3)
N5—N6—C18	114.2 (3)	C16—C17—H17	119.0
N8—N7—C29	114.6 (3)	C12—C17—H17	119.0
N7—N8—C31	113.5 (3)	C23—C18—C19	119.2 (3)
C11—O1—H1A	109.5	C23—C18—N6	123.5 (3)
C13—O3—H3A	109.5	C19—C18—N6	117.3 (3)
C24—O4—H4A	109.5	C18—C19—C20	120.0 (4)
C26—O6—H6	109.5	C18—C19—H19	120.0
C2—C1—H1B	109.5	C20—C19—H19	120.0
C2—C1—H1C	109.5	C21—C20—C19	120.0 (4)
H1B—C1—H1C	109.5	C21—C20—H20	120.0
C2—C1—H1D	109.5	C19—C20—H20	120.0
H1B—C1—H1D	109.5	C20—C21—C22	119.5 (4)
H1C—C1—H1D	109.5	C20—C21—H21	120.3
N1—C2—C3	109.6 (3)	C22—C21—H21	120.3
N1—C2—C1	119.3 (3)	C21—C22—C23	120.7 (4)
C3—C2—C1	131.1 (3)	C21—C22—H22	119.6
C4—C3—C2	106.7 (3)	C23—C22—H22	119.6
C4—C3—H3B	126.6	C18—C23—C22	120.5 (4)
C2—C3—H3B	126.6	C18—C23—H23	119.7
N2—C4—C3	106.3 (3)	C22—C23—H23	119.7
N2—C4—C5	121.4 (3)	O5—C24—O4	123.6 (3)
C3—C4—C5	132.3 (3)	O5—C24—C25	121.4 (3)
C4—C5—H5A	109.5	O4—C24—C25	115.0 (3)
C4—C5—H5B	109.5	C30—C25—C26	118.6 (3)
H5A—C5—H5B	109.5	C30—C25—C24	121.0 (3)
C4—C5—H5C	109.5	C26—C25—C24	120.3 (3)
H5A—C5—H5C	109.5	O6—C26—C27	117.8 (3)
H5B—C5—H5C	109.5	O6—C26—C25	122.5 (3)
C7—C6—H6A	109.5	C27—C26—C25	119.7 (3)
C7—C6—H6B	109.5	C28—C27—C26	121.0 (3)
H6A—C6—H6B	109.5	C28—C27—H27	119.5
C7—C6—H6C	109.5	C26—C27—H27	119.5
H6A—C6—H6C	109.5	C27—C28—C29	120.0 (3)
H6B—C6—H6C	109.5	C27—C28—H28	120.0
N3—C7—C8	106.5 (3)	C29—C28—H28	120.0
N3—C7—C6	121.8 (3)	C30—C29—C28	119.0 (3)
C8—C7—C6	131.7 (3)	C30—C29—N7	115.7 (3)
C7—C8—C9	107.0 (3)	C28—C29—N7	125.3 (3)
C7—C8—H8	126.5	C29—C30—C25	121.6 (3)
C9—C8—H8	126.5	C29—C30—H30	119.2
N4—C9—C8	109.6 (3)	C25—C30—H30	119.2
N4—C9—C10	120.2 (3)	C36—C31—C32	119.4 (3)
C8—C9—C10	130.2 (3)	C36—C31—N8	115.9 (3)
C9—C10—H10A	109.5	C32—C31—N8	124.8 (3)
C9—C10—H10B	109.5	C33—C32—C31	120.4 (3)
H10A—C10—H10B	109.5	C33—C32—H32	119.8
C9—C10—H10C	109.5	C31—C32—H32	119.8
H10A—C10—H10C	109.5	C34—C33—C32	119.7 (4)
H10B—C10—H10C	109.5	C34—C33—H33	120.1
O2—C11—O1	124.0 (3)	C32—C33—H33	120.1
O2—C11—C12	121.9 (3)	C33—C34—C35	120.2 (3)
O1—C11—C12	114.1 (3)	C33—C34—H34	119.9
C17—C12—C13	118.5 (3)	C35—C34—H34	119.9
C17—C12—C11	121.6 (3)	C34—C35—C36	120.6 (4)
C13—C12—C11	119.9 (3)	C34—C35—H35	119.7
O3—C13—C14	118.4 (3)	C36—C35—H35	119.7
O3—C13—C12	122.0 (3)	C35—C36—C31	119.6 (3)
C14—C13—C12	119.6 (3)	C35—C36—H36	120.2
C15—C14—C13	121.1 (3)	C31—C36—H36	120.2
			
C2—N1—N2—C4	−0.6 (3)	N5—N6—C18—C23	−13.3 (5)
C7—N3—N4—C9	−0.3 (4)	N5—N6—C18—C19	166.9 (3)
C16—N5—N6—C18	178.9 (3)	C23—C18—C19—C20	0.0 (6)
C29—N7—N8—C31	179.7 (2)	N6—C18—C19—C20	179.9 (3)
N2—N1—C2—C3	0.3 (3)	C18—C19—C20—C21	−0.3 (6)
N2—N1—C2—C1	−179.6 (3)	C19—C20—C21—C22	1.0 (7)
N1—C2—C3—C4	0.1 (4)	C20—C21—C22—C23	−1.4 (8)
C1—C2—C3—C4	180.0 (3)	C19—C18—C23—C22	−0.4 (7)
N1—N2—C4—C3	0.7 (3)	N6—C18—C23—C22	179.7 (4)
N1—N2—C4—C5	−179.4 (3)	C21—C22—C23—C18	1.1 (8)
C2—C3—C4—N2	−0.5 (4)	O5—C24—C25—C30	174.8 (3)
C2—C3—C4—C5	179.6 (3)	O4—C24—C25—C30	−7.0 (5)
N4—N3—C7—C8	0.3 (4)	O5—C24—C25—C26	−9.7 (5)
N4—N3—C7—C6	179.1 (3)	O4—C24—C25—C26	168.5 (3)
N3—C7—C8—C9	−0.1 (4)	C30—C25—C26—O6	−178.9 (3)
C6—C7—C8—C9	−178.8 (3)	C24—C25—C26—O6	5.5 (5)
N3—N4—C9—C8	0.2 (4)	C30—C25—C26—C27	3.1 (5)
N3—N4—C9—C10	179.7 (3)	C24—C25—C26—C27	−172.5 (3)
C7—C8—C9—N4	−0.1 (4)	O6—C26—C27—C28	179.2 (3)
C7—C8—C9—C10	−179.5 (4)	C25—C26—C27—C28	−2.8 (5)
O2—C11—C12—C17	−179.3 (3)	C26—C27—C28—C29	0.3 (5)
O1—C11—C12—C17	−0.1 (4)	C27—C28—C29—C30	1.8 (5)
O2—C11—C12—C13	−1.1 (5)	C27—C28—C29—N7	−179.5 (3)
O1—C11—C12—C13	178.1 (3)	N8—N7—C29—C30	178.8 (3)
C17—C12—C13—O3	−179.6 (3)	N8—N7—C29—C28	0.0 (4)
C11—C12—C13—O3	2.2 (5)	C28—C29—C30—C25	−1.4 (5)
C17—C12—C13—C14	2.0 (5)	N7—C29—C30—C25	179.7 (3)
C11—C12—C13—C14	−176.2 (3)	C26—C25—C30—C29	−1.0 (5)
O3—C13—C14—C15	179.1 (3)	C24—C25—C30—C29	174.5 (3)
C12—C13—C14—C15	−2.5 (5)	N7—N8—C31—C36	172.8 (3)
C13—C14—C15—C16	0.8 (6)	N7—N8—C31—C32	−7.7 (4)
C14—C15—C16—C17	1.3 (5)	C36—C31—C32—C33	−1.7 (5)
C14—C15—C16—N5	179.4 (3)	N8—C31—C32—C33	178.8 (3)
N6—N5—C16—C17	176.9 (3)	C31—C32—C33—C34	1.3 (6)
N6—N5—C16—C15	−1.2 (5)	C32—C33—C34—C35	−0.6 (6)
C15—C16—C17—C12	−1.7 (5)	C33—C34—C35—C36	0.4 (6)
N5—C16—C17—C12	−180.0 (3)	C34—C35—C36—C31	−0.7 (6)
C13—C12—C17—C16	0.1 (5)	C32—C31—C36—C35	1.4 (5)
C11—C12—C17—C16	178.3 (3)	N8—C31—C36—C35	−179.1 (3)

### Hydrogen-bond geometry (Å, °)

**Table:**

Cg1 and Cg2 are the centroids of the C31–C36 and C25–C30 rings, respectively.

**Table:**

*D*—H···*A*	*D*—H	H···*A*	*D*···*A*	*D*—H···*A*
O6—H6···O2^i^	0.82	2.43	3.019 (3)	129.
O6—H6···O5	0.82	1.89	2.605 (3)	145.
O4—H4A···N4^ii^	0.82	1.75	2.564 (3)	169.
O3—H3A···O5^iii^	0.82	2.45	3.019 (3)	128.
O3—H3A···O2	0.82	1.85	2.574 (3)	147.
O1—H1A···N1^iv^	0.82	1.75	2.565 (3)	171.
N3—H3···O3^v^	0.86	2.17	3.008 (3)	165.
N2—H2···O6^vi^	0.86	2.15	3.002 (3)	170.
C1—H1D···Cg1^vii^	0.96	2.75	3.677 (4)	164
C5—H5B···Cg2^vii^	0.96	2.96	3.840 (4)	153

Symmetry codes: (i) *x*, *y*−1, *z*−1; (ii) −*x*+2, −*y*+1, −*z*; (iii) *x*, *y*+1, *z*+1; (iv) *x*, *y*, *z*+1; (v) −*x*+2, −*y*+2, −*z*+1; (vi) *x*, *y*+1, *z*; (vii) −*x*+1, −*y*+1, −*z*+1.
